# Treatment of Endometriosis with the GnRHa Deslorelin and Add-Back Estradiol and Supplementary Testosterone

**DOI:** 10.1155/2015/934164

**Published:** 2015-12-31

**Authors:** Sanjay K. Agarwal, AnnaMarie Daniels, Steven R. Drosman, Laurence Udoff, Warren G. Foster, Malcolm C. Pike, Darcy V. Spicer, John R. Daniels

**Affiliations:** ^1^Center for Endometriosis Research and Treatment, Department of Reproductive Medicine, UC San Diego, 9500 Gilman Drive, No. 0633, La Jolla, CA 92093-0633, USA; ^2^Balance Pharmaceuticals, 842 N Las Casas Avenue, Pacific Palisades, CA 90272, USA; ^3^Genesis Center for Clinical Research, 3651 4th Avenue, San Diego, CA 92103, USA; ^4^Genetics and IVF Institute, 3015 Williams Drive, Fairfax, VA 22031, USA; ^5^Department of Obstetrics and Gynecology, McMaster University, 1280 Main Street West, HSC-3N52D, Hamilton, ON, Canada L8S 4K1; ^6^Department of Preventive Medicine, University of Southern California, 1441 Eastlake Avenue, Los Angeles, CA 90033, USA; ^7^Division of Medical Oncology, University of Southern California, 1441 Eastlake Avenue, Los Angeles, CA 90033, USA

## Abstract

*Background*. This randomized, multicenter, open-label clinical trial was intended to generate pilot data on the efficacy and safety of the gonadotropin-releasing hormone agonist (GnRHa) deslorelin (D) with low-dose estradiol ± testosterone (E_2_  ± T) add-back for endometriosis-related pelvic pain.* Methods*. Women with pelvic pain and laparoscopically confirmed endometriosis were treated with a six-month course of daily intranasal D with concurrent administration of either transdermal E_2_, intranasal E_2_, or intranasal E_2_  + T. Efficacy data included evaluation of dyspareunia, dysmenorrhea, pelvic pain, tenderness, and induration. Cognition and quality of life were also assessed. Safety parameters included assessment of endometrial hyperplasia, bone mineral density (BMD), and hot flashes.* Results*. Endometriosis symptoms and signs scores decreased in all treatment arms from a baseline average of 7.4 to 2.5 after 3 months of treatment and 3.4 after 6 months. BMD changes and incidence of hot flashes were minimal, and no endometrial hyperplasia was observed. Patient-reported outcomes showed significant improvement across multiple domains.* Conclusions*. Daily intranasal D with low dose E_2_  ± T add-back resulted in significant reduction in severity of endometriosis symptoms and signs with few safety signals and minimal hypoestrogenic symptoms that would be expected with the use of a GnRHa alone.

## 1. Introduction

Endometriosis is the presence of endometrial tissue external to the uterus [1]. Up to 11% of women have endometriosis, although many may not reach a clinical threshold of severity for diagnosis [2]. Endometriosis is frequently associated with significant pelvic pain [3] and, like many chronic pain conditions, can lead to deterioration in quality of life. The ectopic endometrial cells demonstrate hormonal responsiveness similar to native endometrium. Without treatment, relief of endometriosis is typically experienced only with the reduction of circulating estrogens as occurs at menopause. Current treatment options include surgical tissue fulguration or excision and hormonal manipulations [3].

Endometriosis lesions atrophy with interventional suppression of sex steroids [3]. This can be achieved with chronic administration of a gonadotropin-releasing hormone agonist (GnRHa), which produces paradoxical suppression of follicle-stimulating hormone and luteinizing hormone release, and hence suppression of gonadal function. GnRHa usage markedly reduces normal ovarian production of estradiol (E_2_) and testosterone (T) and lowers the circulating levels of these hormones. Despite its benefits in reducing pelvic pain and other symptoms of endometriosis, GnRHa usage must be discontinued after a period of time, typically 12 months, because of concerns over loss of bone mineral density (osteoporosis). Therapeutic outcomes may persist for some time after the GnRHa is discontinued. GnRHa treatment of endometriosis is well established and has been reviewed extensively [3–10]. Currently, leuprolide, nafarelin, and goserelin are widely used in USA and others are available worldwide. Deslorelin (D), a potent GnRHa, has not previously been studied for endometriosis.

It is generally not considered appropriate to treat with a GnRHa alone because of a substantial reduction in bone mineral density (BMD), significant symptoms of estrogen deficiency such as hot flashes [11, 12], and sexual complaints such as the loss of libido and vaginal dryness. The use of add-back sex steroids with GnRH analogues is aimed at preventing the negative effects of the GnRHa while maintaining an effective therapy for endometriosis [13–19]. Norethindrone acetate at 5 mg/day is the only current FDA-approved add-back [13]. This high dose of progestin add-back is not universally tolerated and may have other risks associated with its use; however, and for this reason, some women taking GnRH agonists prefer to accept the hypoestrogenic side effects of the GnRH agonists rather than take the add-back. This represents a shortfall in endometriosis intervention options.

The BMD loss seen in GnRHa treatment is the result of the pharmacologic reduction in circulating E_2_ levels [20]. For this reason, the evaluation of E_2_ as an add-back would be logical. However, endometrial hyperplasia is a recognized consequence of unopposed E_2_ use in postmenopausal women with the incidence related to the dose and duration of use [21, 22]. Another concern regarding the use of E_2_ add-back is that if the administered dose is too high, it may decrease the efficacy of the GnRHa. Conversely, if the E_2_ add-back dosage is too low, loss of BMD occurs. To date, there are limited prospective data available regarding the use of estrogen alone as add-back [23]. It is not known if endometrial hyperplasia results from a low dose of unopposed E_2_ add-back over a 6-month period in premenopausal women receiving a GnRH agonist.

Sexual function, including libido, is influenced by circulating T titers [24], a sex steroid normally secreted by the ovary [25]. T also maintains BMD [26]. GnRH agonists, including D, have been shown to reduce serum T levels in premenopausal women [27]. For this reason, the evaluation of T as an add-back would be logical. Androgen replacement has received minimal attention in women. Combinations of oral estrogen with methyltestosterone are used as a form of menopausal hormone replacement therapy and combined estrogen and T pellets have been evaluated and used outside the United States as postmenopausal hormone replacement therapy [28–31]. Another putative benefit of including T into the add-back regimen would be that it is expected to reduce the dose of E_2_ that would be required for add-back, thereby further lowering the risk of endometrial hyperplasia.

In this study, D was used for the treatment of endometriosis-related pain. Because the usage of this GnRHa would otherwise be compromised by its hypoestrogenic side effects, of which a decrease in BMD is the most concerning, add-back strategies were used. To achieve a dosage balance between a possible flare of endometriosis symptoms and ineffective prevention of BMD loss [23], a low dose of E_2_ was employed. The E_2_ was delivered in two different forms in order to explore alternative routes of administrations; E_2_ in a transdermal patch and in an intranasal spray was employed. Additionally, in a subgroup, T was used in addition to E_2_ in an intranasal spray. No prior study has evaluated the use of low dose E_2_ plus T as add-back during the GnRHa treatment of endometriosis with D. The purpose of this study was to assess the use of low dose E_2_  ± T as add-back regimens with the use of D for the treatment of endometriosis. Our hypothesis was that these treatments would result in relief of endometriosis symptoms and concomitant improvement in quality of life, with low rates of BMD loss and other hypoestrogenic symptoms, specifically hot flashes.

## 2. Methods

### 2.1. Subjects

Subjects were recruited from investigators' practices at four US sites (2 academic and 2 private practice) between 2000 and 2001. The study was approved by the local institutional review boards and each subject gave written informed consent before study activities began.

Subjects were premenopausal women between 25 and 45 who were nonsmokers, in good health, and had regular menstrual cycles. Subjects had endometriosis that had been laparoscopically confirmed within 3 years of study entry; dysmenorrhea, dyspareunia, and/or nonmenstrual pelvic pain (NMPP) of moderate or severe intensity; and lumbar spine BMD of at least 85% of normal for their age.

Subjects were excluded for pelvic pain of nonendometriosis origin, polycystic ovarian syndrome, current or recent significant medical conditions that might interfere with diagnosis, treatment or interpretation of outcome measures or malignancy, or contraindications to using nasal spray or estrogen-containing medications. Women were also excluded if they were using any hormonal medications or other medications for osteoporosis.

### 2.2. Intervention

All subjects were treated with 1 mg/day intranasal D acetate as a single daily 100 *μ*L nasal spray. Before the start of treatment, subjects were assigned to one of three groups for add-back hormones. Group assignments were sequentially allocated centrally by the study sponsor. Thus, subjects were treated with one of three hormone add-back regimens: 50 *μ*g/day E_2_ via a transdermal patch (D + E_2_ transdermal); 300 *μ*g/day E_2_ via a single daily 100 *μ*L nasal spray (D + E_2_ nasal); or 300 *μ*g/day E_2_ combined with 275 *μ*g/day T via a single daily 100 *μ*L nasal spray (D + E_2_ + T nasal). Group assignment was not blinded.

In this exploratory study, add-back dosages were used based on published regimens. A 50 *μ*g per day transdermal E_2_ patch has been reported to be effective in preserving bone in most postmenopausal women [32–34]. The level of E_2_ suppression seen in women treated with D in previous studies is similar to that reported in postmenopausal women; thus, a 50 *μ*g per day transdermal dose of E_2_ was expected to preserve bone in this D-treated population. The 300 *μ*g/day dose (confirmed by serum pharmacokinetics) of E_2_ in the nasal spray was estimated to be slightly lower than that delivered by the 50 *μ*g per day transdermal patch. The 275 *μ*g/day dose of T in the nasal spray was calculated to replace that lost from ovarian production [35].

### 2.3. Study Design and Outcome Measures

This study primarily aimed to demonstrate whether D treatment reduced pelvic pain associated with endometriosis. Information was also sought regarding quality of life and the control of hypoestrogenic symptoms (hot flashes, vaginal dryness, and libido loss). The possibility of E_2_ inducing unopposed proliferation of the endometrium was assessed via biopsies. Another key endpoint was to determine whether, and to what extent, E_2_ transdermal, E_2_ nasal, and E_2_ + T nasal add-back prevented the BMD loss that has been associated with the use of GnRHa alone.

#### 2.3.1. Screening and Baseline Phase

Subjects completed a daily diary for two 28-day periods (i.e., two menstrual cycles) prior to baseline measurements. The diary recorded vaginal bleeding (rated as none, spotting, minimal, moderate, or severe), severity of pain on a standard 10 cm visual analogue scale (VAS), pain medication usage (over-the-counter analgesics excluded), and the severity of any hot flashes (rated as mild, moderate, or severe).

Baseline assessments included endometrial biopsy, laboratory blood testing (complete blood count, chemistry panel with lipids, and serum hormone panel [E_2_, T, progesterone]), and BMD of the lumbar spine (L1–L4) via dual-energy X-ray absorptiometry (DEXA [36]). Patient-reported outcomes included assessment of pelvic symptoms via the Combined Pelvic Symptom and Sign Score (CPSSS [37]), quality of life via the SF-36 scale [38], and an assessment of sexual functioning and everyday problems via a modified version of the Breast Cancer Prevention Trial Symptom Checklist previously developed for the National Surgical Adjuvant Breast and Bowel Project breast cancer prevention trials [39]. Because memory impairment has been reported with GnRH agonist treatment [40], cognitive function was monitored via the Memory Observation Questionnaire (MOQ [41]).

#### 2.3.2. Treatment Phase

After baseline measurements, subjects began daily treatment with D and their assigned add-back hormone regimen. Treatment continued for 6 months. During this time, subjects continued to make daily diary entries. Subjects made clinic visits after 3 and 6 months, during which the baseline patient-reported outcomes were repeated. Laboratory blood values and BMD assessment were repeated at 6 months, and an endometrial biopsy was performed. Safety events were recorded throughout the study and were managed by the investigators according to institutional protocols.

### 2.4. Data Management and Analysis

Data were gathered on preprinted case report forms or diary sheets. Data quality and compliance with study procedures were confirmed by regular site monitoring visits.

Diaries were divided into 28-day intervals and data were summed within each interval for each subject. For any 28-day interval, the number of bleeding days and the number of pain medication usage days could range from 0 to 28, and the pain score could range from 0 to 280 (i.e., 0–10 cm VAS on each day, summed over 28 days). The cumulative number of hot flashes in each severity category was recorded. Baseline diary data results were the average of the two 28-day intervals during the screening phase. Diary data during the 28-day interval preceding the 3-month and 6-month clinic visits were used during the treatment phase for comparison to baseline.

Quantitative baseline and follow-up assessments were obtained using standard techniques (DEXA radiographic technology in the case of BMD scans and histological examination for evidence of hyperplasia in endometrial biopsies).

Patient-reported outcomes at baseline and follow-ups were scored according to the tools' standard instructions. For the SF-36 and other quality of life scales, domain values were converted to a 0–100 scale, with 100 indicating highest quality of life.

Safety data were expressed as the cumulative frequency of study/intervention-related adverse events. Safety signals also included the MOQ (in which domain values were converted to a 0–10 scale, with 10 indicating best cognitive performance), laboratory values, and endometrial biopsies.

Unless otherwise noted, all descriptive data are presented as means ± standard error of the mean (SEM). For outcomes regarding endometriosis symptoms, hypoestrogenic symptoms, quality of life, and safety, because there was no statistical difference between groups, data were combined across all three treatment groups. For the BMD outcomes, results for the three groups are presented separately. Hypothesis testing used one-way ANOVA with appropriate post hoc comparisons or Wilcoxon signed-rank tests with significance levels set at *p* < 0.05. All analyses were carried out by using StatView 5 (Abacus Corporation, Berkeley, CA). 

## 3. Results

### 3.1. Subject Demographics and Baseline Characteristics

41 individuals were screened for the study. Of these, 26 proceeded to the treatment phase. Data from six subjects could not be included in the final analysis because four discontinued treatment and two had confounding comorbidities during treatment (major surgeries for unrelated issues). Thus, 20 subjects were included in the analysis. There were 5 subjects in the D + E_2_ transdermal group, 7 in the D + E_2_ nasal group, and 8 in the D + E_2_ + T nasal group.

The average age at study entry was 36.6 years (±1.1; range 26.9–45.1). Most subjects were Caucasian (*n* = 15; 75%) or African-American (*n* = 3; 15%). The average body weight at study entry was 72.8 kg (±3.8; range 48.2–107.7), with an initial BMI of 26.6 kg/m^2^ (±1.4; range 19.1–41.9). There were no significant differences between the treatment groups for these measures.

### 3.2. Clinical Efficacy: Endometriosis Symptoms

At baseline, subjects recorded bleeding (greater than spotting) on 5.2 out of every 28 days (18.6%). After three months of treatment, most subjects were amenorrheic, with an average of 0.8 bleeding days per 28 (2.8%; *p* < 0.0001). Some subjects had occasional bleeding after six months of treatment, although the average incidence of bleeding was halved relative to baseline (2.5 bleeding days per 28; 8.9%; *p* > 0.05).

Overall pain was reduced from 58.1 out of a possible 280 over the 28-day interval at baseline to 17.5 at 3 months and 19.6 at 6 months (both *p*s < 0.0001). Pain on bleeding days was also markedly reduced: at baseline, pain was rated as 23.6 out of a possible 52 across 5.2 bleeding days. At 3 months, pain was completely eliminated—a rating of 0 by all subjects—during bleeding days, and at 6 months, pain was rated as 2.6 out of a possible 25 across 2.5 bleeding days (*p*s < 0.0001). Similarly, the number of days on which pain medications other than acetaminophen or salicylates were used decreased from 6.7 out of every 28 days at baseline to 0.7 and 1.1 days out of every 28 after 3 and 6 months of treatment, respectively (*p*s < 0.0002; see Table 1).

Total scores on the CPSSS were 7.4 at baseline and reduced to 2.5 after 3 months of treatment and to 3.4 after 6 months of treatment (*p*s < 0.0001). All of the individual CPSSS parameters showed statistically significant improvements, particularly pelvic pain and dysmenorrhea (see Table 1).

Across 17 subjects, menses returned an average of 39.5 days (range 24–69 days) after the last treatment day. Two subjects remained amenorrheic at their last follow-up (84 and 181 days after the last treatment day), and one subject was lost to follow-up following completion of drug treatment.

### 3.3. Clinical Efficacy: BMD Preservation

Baseline BMD for all subjects was 1.119 g/cm^2^ (range 0.871–1.375). For all subjects, the mean BMD after 6-months of treatment was 99.3% of baseline values (*p* > 0.05). Subjects treated with D + E_2_ nasal retained 99.6% (±1.1) of their BMD (*p* > 0.05), those treated with D + E_2_ transdermal retained 97.8% (±1.0) of their BMD (*p* = 0.04), and those treated with D + E_2_ + T nasal retained 99.9% (±0.7) of their BMD (*p* > 0.05).

### 3.4. Clinical Efficacy: Quality of Life and Hypoestrogenic Symptoms

There were statistically significant improvements relative to baseline for five of the ten quality of life domains: physical functioning, role physical, bodily pain, social functioning, and vitality; those that were unaffected by treatment were already within normative ranges for women of similar age at baseline [42]. Quality of life issues with everyday problems was significantly improved with treatment, and sexual functioning outcomes were unaffected (see Table 2).

**Figure 1:**
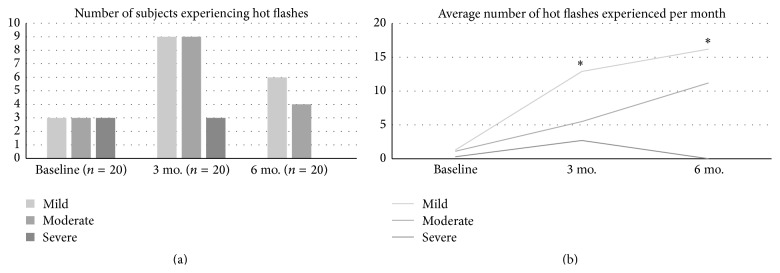
Fewer than half of subjects experienced hot flashes with D with E_2_  ± T treatment; severe hot flashes were not present after 6 months of treatment (a). On average, mild hot flashes occurred more frequently than moderate or severe hot flashes; severe hot flashes were not present after 6 months of treatment (b). *∗* indicates a statistically significant difference relative to baseline values, *p* ≤ 0.05.

At baseline, few subjects (15%) experienced hot flashes of any severity. During treatment, the proportion of subjects who experienced mild or moderate hot flashes temporarily increased, although the majority of subjects never experienced hot flashes at any time before or during treatment (see Figure 1(a)). For those subjects who experienced hot flashes, the average number of mild hot flashes experienced per month increased during treatment (all *p*s < 0.05), but the incidence of moderate and severe hot flashes was not significantly increased (see Figure 1(b)). No severe hot flashes were recorded after 6 months of treatment.

### 3.5. Safety Measures

D treatment with E_2_  ± T add-back was safe. Treatment largely did not negatively impact cognition, although an approximately 15% of subjects reported a decrease in concentration after six months of treatment. White blood count and hemoglobin levels were unchanged during the 6-month treatment course. There appeared to be a 9.5% decrease in platelet count, which was statistically significant, but the platelet count remained well within normal range for all subjects. Cholesterol, HDL, and LDL values were unchanged throughout treatment (*p*s > 0.05). Average VLDL increased by 22.6% and triglycerides increased by 22.7% (*p*s < 0.05), although these values remained within normal ranges for all subjects. Crucially, no instances of endometrial hyperplasia were identified after 6 months of drug treatment. Endometrial tissue was proliferative in all evaluable cases (see Table 3).

There were no adverse events (AEs) that were classified by the investigators as definitely related to treatment. There was one AE, mild leg cramps, that was classified as probably related to treatment. There were 17 AEs possibly related to treatment (most commonly headache, symptoms of the nose [from the nasal spray], and acne), and four AEs considered remotely related to treatment. All AEs were expected and resolved. In addition, there were two serious adverse events (SAEs) in subjects who ultimately withdrew from the study. Neither were related to the study treatment; both cases involved intercurrent surgery for nonendometriosis conditions (see Table 4).

## 4. Discussion

This pilot study is the first report of the GnRH agonist D used for the treatment of endometriosis in conjunction with low-dose E_2_ and T as hormonal add-back. The initial findings suggest that this regimen is a viable intervention. The evaluated subjects were representative of typical endometriosis patients [43]. Prior to the study treatment, the subjects experienced endometriosis symptoms that caused considerable pain and negatively impacted on quality of life. Treatment with 1 mg/day of intranasal D acetate with E_2_  ± T reduced bleeding and induced amenorrhea in the majority of subjects. Treatment significantly relieved pain and other symptoms of endometriosis, including dysmenorrhea and pelvic induration. GnRHa is widely recognized as effective for the pain of endometriosis [44], and add-back adjuvant hormone therapy can reduce side effects and improve quality of life outcomes relative to GnRHa treatment alone [45, 46]. It is notable that only two subjects out of 26 remained amenorrheic 3–6 months after stopping the treatment, suggesting that no overall concern for fertility would be expected with this treatment [47].

Clinical efficacy was achieved without inducement of hypoestrogenic symptoms, presumably because of the add-back hormones. Specifically, the majority of subjects did not experience newly developed hot flashes with the treatment (and those that did experienced only mild hot flashes), suggesting that the add-back hormone regimen was sufficient [48, 49]. It is thought that the T add-back could be especially beneficial for alleviating GnRHa-related symptoms of vaginal dryness and libido loss, similar to findings in studies of female sexual dysfunction [50, 51].

There appeared to be a slight decrement in the self-reported treatment benefits at the 6-month time point relative to the 3-month time point. However, hypoestrogenic symptoms increased between 3 and 6 months. Because the hypoestrogenic symptoms provide better empirical evidence of the treatment effect (i.e., blocking of sex steroid production), the apparent reduction in pain relief may be a psychological phenomenon related to accommodation due to the passage of time.

Add-back hormones were effective in preventing the BMD loss that would have otherwise been seen with D treatment alone. At baseline, all subjects had BMD 85% of age-adjusted norms (or better). BMD was maintained during treatment, with the proportion of 99.3% of baseline BMD remaining after 6 months of treatment. Of particular note is that the overall change in mean BMD with 6-month treatment in the current study (−0.7%) is similar to that reported with 6-month treatment with leuprolide acetate with 5 mg norethindrone acetate add-back (−1.3% [13]). Transdermal E_2_ add-back was the least effective in preserving BMD with 2.2% loss over 6 months of treatment. E_2_ + T nasal spray resulted in the best outcomes, notwithstanding the similarity of outcomes with all add-back regimens in this study. The treatment-related safety events in this study were as expected for this type of treatment [52] and were usually mild.

Crucially, endometrial tissue was proliferative in all evaluable cases and no instances of endometrial hyperplasia were observed. This is in contrast to the conclusions of a recent systematic review, in which unopposed estrogen was associated with proliferation of endometrial tissue and endometrial hyperplasia [53]. As a result, estrogen therapy in combination with progestogen has become standard. The evidence supporting this widespread practice change, however, is based on results in postmenopausal women for whom quiescent hormonal metabolism and the concomitant responsiveness to exogenous therapy differs from that of women of reproductive potential. Estrogen-alone therapy has been safely tolerated by premenopausal women in recent studies of its short-term application [54, 55]. Thus, more research regarding this question may be needed.

D has not been tested for endometriosis symptoms before this study. Primarily used in veterinary fertility applications, it may be a preferable alternative to other GnRHa options in humans because it requires a low dose, and slow-release preparations are available [56]. The addition of low-dose E_2_ was effective in preventing GnRHa-associated BMD loss. Intranasal administration of E_2_ appeared to be preferable to transdermal administration in terms of BMD preservation. This may occur because of differences in bioavailability [57]. The addition of add-back T along with E_2_ had an apparent additive effect on maintaining BMD. It may also have a beneficial effect on vaginal dryness and libido, as has been reported in the literature [58].

The interpretability of this study is limited by its pooling of outcomes regarding endometriosis symptoms, quality of life, and hypoestrogenic symptoms across all treatment groups. This was done to compensate for the small sample size (itself another study limitation albeit common in pilot work) and to avoid obscuring the main effect through analysis in small subgroups.

## 5. Conclusions

In summary, this clinical trial is the first study to demonstrate that add-back comprising low dose E_2_ with or without T can be used to relieve endometriosis symptoms and to protect from hypoestrogenic symptoms and BMD loss seen with D, a GnRHa. Safety profiles were favorable. Larger randomized studies are required to confirm these encouraging initial findings.

## Figures and Tables

**Table 1:** D with E_2_ ± T add-back reduced endometriosis symptoms. Values are means (SEM); *∗* indicates a statistically significant difference relative to baseline values, *p* ≤ 0.05.

	Baseline *N* = 20	3 months *N* = 20	6 months *N* = 20
Number of days out of 28 with bleeding greater than spotting	5.2 (0.3)	0.8 (0.5)^*∗*^	2.5 (1.4)
Overall pain rating, out of 28	58.1 (8.2)	17.5 (4.9)^*∗*^	19.6 (5.6)^*∗*^
Pain rating on bleeding days	23.6 (3.2) *out of 52*	0.0 (0.0)^*∗*^ *out of 8*	2.6 (1.3)^*∗*^ *out of 25*
Number of days out of 28 on which pain medication was used	6.4 (1.0)	0.7 (0.4)^*∗*^	1.1 (0.5)^*∗*^
CPSSS total	7.4 (0.4)	2.5 (0.5)^*∗*^	3.4 (0.7)^*∗*^
NMPP	1.6 (0.1)	0.6 (0.1)^*∗*^	0.8 (0.2)^*∗*^
Dysmenorrhea	2.1 (0.2)	0.3 (0.1)^*∗*^	0.5 (0.2)^*∗*^
Dyspareunia	1.2 (0.2)	0.5 (0.2)^*∗*^	0.8 (0.2)^*∗*^
Pelvic tenderness	1.5 (0.1)	0.8 (0.2)^*∗*^	0.9 (0.2)^*∗*^
Pelvic induration	1.2 (0.1)	0.4 (0.1)^*∗*^	0.6 (0.2)^*∗*^

**Table 2:** D with E_2_ ± T add-back improved quality of life. Values are means (SEM); *∗* indicates a statistically significant difference relative to baseline values, *p* ≤ 0.05.

	Baseline *N* = 20	3 months *N* = 20	6 months *N* = 20
SF-36	—	—	—
Physical functioning	87.3 (3.2)	98.5 (0.9)^*∗*^	98.0 (1.1)^*∗*^
Role physical	62.5 (9.2)	97.5 (1.7)^*∗*^	92.5 (4.1)^*∗*^
Bodily pain	48.5 (4.3)	84.0 (4.0)^*∗*^	82.5 (4.2)^*∗*^
General health	70.9 (4.5)	76.6 (3.1)	74.3 (4.0)
Social functioning	71.9 (5.5)	88.8 (3.4)^*∗*^	88.1 (4.8)^*∗*^
Vitality	47.5 (5.0)	59.0 (2.9)^*∗*^	63.8 (4.7)^*∗*^
Role emotional	80.0 (7.8)	78.3 (8.1)	86.7 (6.6)
Mental health	69.6 (3.9)	71.8 (4.2)	72.4 (4.1)
Quality of life scales	—	—	—
Everyday problems	81.5 (±1.6)	88.0 (±1.4)^*∗*^	85.5 (±2.3)^*∗*^
Sexual functioning	75.8 (±8.1)	75.0 (±9.4)	67.6 (±6.7)

**Table 3:** D with E_2_ ± T add-back had little safety impact on cognition or laboratory parameters and did not cause endometrial hyperplasia. Values are means (SEM) or percentages; *∗* indicates a statistically significant difference relative to baseline values, *p* ≤ 0.05.

	Baseline *N* = 20	3 months *N* = 20	6 months *N* = 20
MOQ total	—	—	—
Concentration	7.6 (0.6)	6.4 (0.6)	6.2 (0.6)^*∗*^
Nonverbal	8.1 (0.3)	7.8 (0.4)	7.5 (0.4)
Prospective	7.8 (0.4)	7.5 (0.5)	7.2 (0.5)
Real world	8.1 (0.4)	8.1 (0.5)	7.3 (0.5)
Superlative	3.2 (0.4)	3.3 (0.4)	2.8 (0.5)
Verbal	7.3 (1.4)	7.0 (0.5)	6.5 (0.5)
Serum lipids	—	—	—
Cholesterol	204.3 (9.4)	—	202.2 (10.0)
HDL	65.2 (2.4)	—	63.6 (3.5)
LDL	121.1 (9.5)	—	115.2 (9.7)
VLDL	18.1 (1.8)	—	23.4 (2.9)^*∗*^
Triglycerides	90.5 (9.2)	—	117.1 (14.6)^*∗*^

	Baseline *N* = 20		6 months *N* = 20

Complete blood count	—	—	—
White blood cells	6.1 (0.4)	—	5.9 (0.4)
Hemoglobin	12.8 (0.2)	—	13.0 (0.2)
Platelets (000)	304 (16.3)	—	275 (16.3)^*∗*^

	Baseline *N* = 17		6 months *N* = 16

Endometrial biopsy	—	—	—
Proliferative	100%	—	100%
Hyperplasia	0%	—	0%
Atrophic	0%	—	0%

**Table 4:** No AEs were reported that could definitely be ascribed to the treatment. In addition to leg cramps, the most common AEs were headache, signs/symptoms of the nose, and acne.

Relationship	Event description	Number of events, based on severity		
		Mild	Moderate	Severe
Definitely related	—			

Probably related	Leg cramps	1		

Possibly related	Headache	2	1	1
	Symptom of the nose	3		
	Acne	1	1	
	Vaginitis infection	1	1	
	Menorrhagia		1	
	Arthralgia	1		
	Dizziness	1		
	Insomnia	1		
	Nausea	1		
	Hot flashes/sweats	1		

Remotely related	Abdominal pain			1
	Peripheral edema		1	
	Symptom of the nose	1		
	Hot flashes/sweats	1		

Unrelated	Surgical procedure needed			2 SAEs
